# Atrial Fibrillation in Diabetes: Epidemiology, Mechanisms and Integrated Management

**DOI:** 10.3390/jcm15135024

**Published:** 2026-06-27

**Authors:** Paschalis Karakasis, Panagiotis Theofilis, Konstantinos Grigoriou, Panagiotis Iliakis, Panayotis K. Vlachakis, Nikolaos Ktenopoulos, Anastasios Apostolos, Anastasios Chatzichidiroglou, Theocharis Koufakis, Antonios P. Antoniadis, Dimitrios Patoulias, Nikolaos Fragakis

**Affiliations:** 1 Second Department of Cardiology, Hippokration General Hospital, Aristotle University of Thessaloniki, Konstantinoupoleos 49, 54642 Thessaloniki, Greece; 2 First Cardiology Department, School of Medicine, Hippokration General Hospital, National and Kapodistrian University of Athens, 11527 Athens, Greecepanayiotisiliakis@gmail.com (P.I.); nikosktenop@gmail.com (N.K.);; 3 Department of Pharmacology, University of Athens, 75 Mikras Asias Avenue, 11527 Goudi, Greece; dinosgrigoriou@gmail.com; 4 Department of Medicine, Division of Cardiology, Angiology and Internal Emergency Medicine, Ruhr University Bochum, Knappschaft Kliniken University Hospital Bochum, 44892 Bochum, Germany; 5 Department of Cardiology, Guy’s and St Thomas’ NHS Foundation Trust, Harefield Hospital, London UB9 6JH, UK; 6 Department of Midwifery, International Hellenic University, 57001 Thessaloniki, Greece; 7 Second Propedeutic Department of Internal Medicine, Faculty of Medicine, School of Health Sciences Aristotle, University of Thessaloniki, 54642 Thessaloniki, Greecedipatoulias@gmail.com (D.P.)

**Keywords:** atrial fibrillation, diabetes mellitus, diabetic atrial cardiomyopathy, cardiometabolic risk, glycaemic variability, integrated management

## Abstract

Atrial fibrillation (AF) and diabetes mellitus frequently coexist and together define a high-risk cardiometabolic phenotype. Diabetes is associated with an increased incidence of AF, although this relationship is strongly influenced by obesity, hypertension, chronic kidney disease (CKD), heart failure (HF), sleep-disordered breathing, and broader metabolic risk clustering. Once AF develops, diabetes is associated with greater thromboembolic and HF risk, impaired quality of life, cognitive vulnerability, and excess mortality. These adverse outcomes may be partly explained by a multidimensional atrial substrate, described here within the conceptual framework of diabetic atrial cardiomyopathy, in which hyperglycaemia, insulin resistance, glycaemic variability, oxidative stress, inflammation, autonomic dysfunction, microvascular disease, lipotoxicity, and epicardial adipose tissue dysfunction may contribute to atrial fibrosis, electrical heterogeneity, impaired calcium handling, mitochondrial injury, and mechanical dysfunction. Collectively, these abnormalities may facilitate AF initiation, persistence, progression, and recurrence after rhythm-control interventions. Management should therefore extend beyond rhythm control and anticoagulation alone. In individuals at increased risk of AF, priorities include cardiometabolic optimization, treatment of obesity, hypertension, CKD, HF, and sleep apnoea, lifestyle intervention, and selective rhythm surveillance. In subclinical AF, decisions regarding anticoagulation should account for AF burden, thromboembolic and bleeding risk, renal function, frailty, and patient preference. In established AF, stroke prevention, symptom-directed rate or rhythm control, cardiometabolic therapy, and longitudinal reassessment remain central. This narrative review integrates the epidemiology, mechanisms, and management of AF in diabetes across the continuum from AF risk to subclinical and clinical disease.

## 1. Introduction

Atrial fibrillation (AF) and diabetes mellitus increasingly converge as two major expressions of contemporary cardiometabolic disease [1]. AF affected an estimated 52.55 million individuals worldwide in 2021 [2,3], while diabetes affected approximately 589 million adults in 2024 and is projected to affect 853 million by 2050 [4], underscoring the expanding population in whom these conditions will coexist. Although diabetes has traditionally been regarded as one component of global cardiovascular risk, accumulating epidemiological, mechanistic, and therapeutic evidence indicates that its relationship with AF is deeper than simple comorbidity overlap.

Diabetes is consistently associated with higher incident AF risk, although this relationship is strongly modulated by obesity, hypertension, CKD, HF, sleep-disordered breathing, renal dysfunction, and broader cardiometabolic risk clustering [5,6]. In established AF, diabetes defines a high-risk phenotype characterized by greater vascular and renal comorbidity, impaired quality of life, increased thromboembolic and HF risk, cognitive vulnerability, and excess mortality [7]. This adverse clinical profile may reflect a multidimensional atrial substrate in which hyperglycaemia, insulin resistance, glycaemic variability, advanced glycation end products, oxidative stress, inflammation, autonomic dysfunction, microvascular disease, lipotoxicity, and epicardial adipose tissue dysfunction may contribute to atrial fibrosis, conduction heterogeneity, impaired calcium handling, ion-channel remodelling, mitochondrial injury, and atrial mechanical dysfunction [8]. Diabetes-related AF should therefore be interpreted as a heterogeneous cardiometabolic phenotype arising from interactions between dysglycaemia and coexisting obesity, hyper-tension, chronic kidney disease (CKD), heart failure (HF), obstructive sleep apnea (OSA), ageing, and other vascular or metabolic stressors, rather than as a uniform disease attributable to diabetes alone.

This conceptual framework has clear implications for clinical practice. Management of AF is no longer confined to controlling ventricular rate or restoring sinus rhythm; increasing attention is now directed toward the cardiometabolic milieu in which the arrhythmia develops and persists. The 2024 ESC AF guideline reflects this broader perspective through the AF-CARE pathway [9], which brings together management of comorbidities and modifiable risk factors, prevention of stroke and systemic thromboembolism, symptom control, and periodic reassessment. The guideline further notes that metformin and SGLT2 inhibitors may be considered when glucose-lowering treatment is required in individuals at risk of AF. Evidence linking adipose tissue to atrial remodelling provides additional support for such a substrate-oriented strategy. In patients undergoing repeat AF ablation, Landra et al. found that left atrial intramyocardial fat was associated with sites of pulmonary vein reconnection, suggesting that localized fatty infiltration may contribute to the persistence or recovery of atrial conduction [10]. Emerging data concerning GLP-1 receptor agonists and co-agonists are also relevant, as these agents may be associated with a lower risk of AF in patients with overweight or obesity [11]. Collectively, these findings suggest that cardiometabolic treatment may influence arrhythmia susceptibility through effects on the atrial substrate as well as through improvements in systemic metabolic health.

The present review brings these strands of evidence together within the concept of diabetic atrial cardiomyopathy. Rather than considering the epidemiology, mechanisms, and treatment of diabetes-associated AF as separate topics, it examines how these domains interact across the course of the disease. The discussion is framed by the 2024 ESC recommendations and organized according to three clinically relevant stages: patients with diabetes who remain at increased risk of AF, those with device-detected subclinical AF, and those with established clinical AF. This structure allows prevention, rhythm surveillance, thromboembolic risk assessment, symptom-directed treatment, cardiometabolic intervention, and follow-up to be considered in relation to the patient’s position along the AF continuum.

## 2. Methods

A comprehensive literature search was conducted in PubMed/MEDLINE, Embase, Scopus, Web of Science, and the Cochrane Library from database inception through 16 June 2026. Major clinical practice guidelines and consensus documents were additionally retrieved from the official repositories of the European Society of Cardiology, American Heart Association, American College of Cardiology, and European Association for the Study of Diabetes. The search strategy combined controlled vocabulary and free-text terms related to “atrial fibrillation”, “diabetes mellitus”, “hyperglycaemia”, “insulin resistance”, “glycaemic variability”, “atrial cardiomyopathy”, “fibrosis”, “inflammation”, “oxidative stress”, “autonomic dysfunction”, “epicardial adipose tissue”, “lipotoxicity”, “anticoagulation”, “rhythm control”, “SGLT2 inhibitors”, and “GLP-1 receptor agonists”. The reference lists of pertinent primary studies, reviews, and guideline documents were also examined to identify additional relevant publications.

For the epidemiological and management sections, eligible evidence comprised peer-reviewed cohort studies, registry analyses, randomized controlled trials, meta-analyses, and authoritative guideline or consensus documents. Preclinical and translational investigations were considered for the mechanistic section when they provided direct insight into diabetes-associated atrial remodelling, electrophysiological disturbance, or AF susceptibility. Conference abstracts, non-peer-reviewed reports, duplicate publications, non-English-language articles, and studies lacking direct relevance to the predefined scope were excluded. Titles, abstracts, and full texts were assessed independently by two authors, with discrepancies resolved through discussion and consensus. Study selection was guided by methodological robustness, clinical relevance, originality, and contribution to the epidemiological, mechanistic, or therapeutic synthesis. Given the narrative design of the review, no formal risk-of-bias assessment or evidence grading was performed.

## 3. Epidemiology

### 3.1. Diabetes and Atrial Fibrillation: Epidemiological Convergence and Clinical Burden

Interpretation of this evidence requires separation of several related but non-equivalent exposures. Prediabetes denotes early dysglycaemia without established diabetes, whereas T1D and T2D differ in underlying biology, age at onset, treatment requirements, and accompanying comorbidity patterns. Diabetes duration reflects cumulative exposure; mean HbA1c estimates average glycaemia; glycaemic variability captures temporal instability; and renal or other microvascular complications indicate end-organ disease that may confer additional AF risk.

Epidemiological evidence consistently supports diabetes as both a risk factor for incident atrial fibrillation (AF) and a major comorbidity shaping AF-related clinical burden [12,13,14]. In the Framingham Heart Study, among 2090 men and 2641 women aged 55–94 years and followed for up to 38 years, diabetes independently predicted AF, with adjusted odds ratios (ORs) of 1.4 in men and 1.6 in women, although its population-level contribution was smaller than that of hypertension, heart failure (HF), or valvular heart disease [15]. This association was subsequently refined in the Atherosclerosis Risk in Communities (ARIC) study, where 13,025 participants were followed through 2007: type 2 diabetes (T2D) was associated with a 35% higher risk of incident AF (hazard ratio [HR] 1.35, 95% confidence interval [CI] 1.14–1.60), and hemoglobin A1c (HbA1c) showed a graded association with AF risk among individuals with diabetes (HR 1.13, 95% CI 1.07–1.20 per 1% increment) [16]. Larger contemporary cohorts indicate that this association extends across earlier stages of dysglycaemia, although it is partly mediated by adiposity and broader cardiometabolic risk clustering. In the Swedish Apolipoprotein Mortality Risk (AMORIS) cohort of 294,057 individuals without baseline cardiovascular disease (CVD), impaired fasting glucose, undiagnosed diabetes, and diagnosed diabetes were associated with progressively higher AF risk over a mean follow-up of 19.1 years, with HRs of 1.19, 1.23, and 1.30, respectively; after adjustment for body mass index (BMI), however, the association persisted only for diagnosed diabetes [17]. Similarly, an umbrella review of 95 meta-analyses found moderate-certainty evidence linking prediabetes with AF and other cardiovascular outcomes, while a UK Biobank analysis of 427,435 participants showed that excess CVD risk across the glycaemic spectrum was substantially attenuated after adjustment for obesity, lifestyle factors, antihypertensive therapy, and statin use [18,19]. Importantly, the attributable contribution of diabetes to AF at the population level may be modest relative to other risk factors: the 2024 AHA statistical update, using ARIC-derived estimates, reported a population-attributable fraction of 3.1% for diabetes mellitus, compared with 21.6% for hypertension and 17.9% for BMI ≥ 25 kg/m^2^ [20]. Accordingly, the relatively modest population-attributable fraction of diabetes does not support universal diabetes-specific AF screening; instead, rhythm surveillance should be concentrated in patients with additional risk enhancers, including advanced age, obesity, hypertension, CKD, HF, albuminuria, OSA, or suggestive symptoms. Taken together, the attenuation of several associations after adjustment for adiposity and other covariates suggests that diabetes may act both as an independent metabolic exposure and as a marker of shared cardiometabolic risk; conventional observational analyses cannot fully separate direct effects from mediation or residual confounding.

The coexistence of diabetes and AF is increasingly common and prognostically important. In the Finnish AntiCoagulation in Atrial Fibrillation (FinACAF) nationwide cohort of 229,565 patients with incident AF, the prevalence of diabetes increased from 15.5% in 2007 to 26.3% in 2018; despite temporal improvements in outcomes, diabetes remained independently associated with ischemic stroke (adjusted incidence rate ratio [IRR] 1.22, 95% CI 1.17–1.26) and mortality (adjusted IRR 1.32, 95% CI 1.29–1.34) [21]. In northeast China, among 18,796 adults aged ≥40 years, AF prevalence was 1.1% overall and increased to 5.0% in those aged ≥80 years; among participants with AF, diabetes was present in 24.2%, yet diabetes control was achieved in only 28.8%, highlighting the frequent coexistence of AF with insufficiently controlled metabolic disease in real-world populations [22]. UK Biobank data further emphasize this clustering, with AF present in 13.68% of individuals with T2D compared with 6.37% of age- and sex-matched individuals without T2D [23]. Evidence in type 1 diabetes (T1D) indicates a distinct but directionally consistent association. In a Swedish prospective case–control study of 36,258 individuals with T1D and 179,980 matched controls, AF occurred in 749 and 2882 individuals, respectively, with adjusted HRs of 1.13 in men and 1.50 in women; risk increased further with poorer glycaemic control and renal complications [24]. A subsequent meta-analysis of four cohort studies estimated a pooled AF risk of 1.30 for T1D, with stronger associations in women and in individuals younger than 65 years, while a Korean nationwide cohort of 20,423,051 adults showed that T1D was associated with higher AF risk than both T2D and no diabetes [25,26]. Once AF is established, diabetes severity appears clinically relevant: in the Anticoagulation and Risk Factors in Atrial Fibrillation (ATRIA) study, diabetes duration ≥3 years was associated with higher ischemic stroke risk among patients with AF (HR 1.74, 95% CI 1.10–2.76), whereas HbA1c categories were not independently predictive; and in a Swedish nationwide AF cohort of 309,611 patients, both T1D and T2D were associated with higher mortality, HF, myocardial infarction, ischemic stroke, and dementia, with consistently larger excess hazards for T1D than T2D [27,28]. Sex-stratified evidence suggests a more complex pattern than the higher absolute AF incidence generally observed in men. In a French nationwide cohort of 2,921,407 adults, the relative association of diabetes with incident AF was stronger in women than in men for both T1D (adjusted HR 1.32 vs. 1.12) and T2D (adjusted HR 1.17 vs. 1.10) [29]. This finding is directionally consistent with the Swedish T1D cohort, in which the excess risk was also greater in women [24], and indicates that absolute and relative risks should be distinguished when characterizing sex differences.

These findings should therefore be interpreted according to diabetes phenotype and the specific exposure measured. Most large population datasets concern T2D, in which insulin resistance, adiposity, hypertension, and cardiorenal comorbidity are prominent; findings in T1D should not be extrapolated directly because earlier onset, absolute insulin deficiency, and longer cumulative exposure may shape risk differently. Within either diabetes type, duration, mean HbA1c, glycaemic variability, and complication burden provide distinct—and not necessarily concordant—information regarding AF susceptibility and prognosis.

### 3.2. Diabetes and Atrial Fibrillation: A High-Risk Cardiometabolic Phenotype

The coexistence of diabetes and AF identifies a high-risk cardiometabolic phenotype in which arrhythmia burden, vascular comorbidity, functional limitation, and neurocognitive vulnerability converge [30,31,32,33,34]. In the Outcomes Registry for Better Informed Treatment of Atrial Fibrillation (ORBIT-AF) [35], diabetes was present in 2874 of 9749 patients with AF (29.5%) and clustered with hypertension, chronic kidney disease (CKD), HF, coronary heart disease, and prior stroke; compared with patients without diabetes, those with diabetes had lower Atrial Fibrillation Effects on Quality of Life scores (80.0 [interquartile range 62.5–92.6] vs. 82.4 [67.6–93.5]) and higher adjusted risks of all-cause mortality, particularly in those aged <70 years (aHR 1.63), as well as cardiovascular mortality (aHR 2.20 in those aged <70 years), hospitalization, cardiovascular hospitalization, non-cardiovascular death, and sudden cardiac death, although diabetes was not associated with higher thromboembolic events, bleeding-related hospitalization, incident HF, or AF progression [35]. The Swiss Atrial Fibrillation (Swiss-AF) study refined this clinical phenotype: among 2411 patients with documented AF, diabetes was not associated with non-paroxysmal AF (OR 1.01), but patients with diabetes less often perceived AF symptoms (OR 0.74), had worse quality of life (β −4.54, 95% CI −6.40 to −2.68), and carried substantially higher odds of hypertension (OR 3.04), myocardial infarction (OR 1.55), HF (OR 1.99), stroke (OR 1.39), and cognitive impairment (OR 1.75) [36]. Contemporary population-screening data further support the concept of silent or under-recognized AF in diabetes: in the Non-invasive Monitoring for Early Detection of Atrial Fibrillation (NOMED-AF) study [37], AF was detected in 25% of older adults with diabetes versus 17% without diabetes, silent AF in 9% versus 7%, and persistent/permanent AF in 12.2% versus 6.9%, using prolonged wearable electrocardiographic monitoring [37]. However, a later NOMED-AF analysis of 881 diabetic participants aged ≥65 years found that diabetes duration [38], treatment regimen, and hemoglobin A1c were not independently associated with AF prevalence, while age and body mass index remained dominant predictors, suggesting that the coexistence of diabetes and AF may reflect cumulative cardiometabolic substrate rather than glycaemia alone [38]. The prognostic implications of new-onset AF in T2D are substantial: in 16,551 UK Biobank participants with T2D free of cardiovascular disease (CVD) and CKD at baseline, incident AF was associated with higher subsequent risks of atherosclerotic cardiovascular disease (hazard ratio [HR] 1.85, 95% CI 1.59–2.16), HF (HR 4.40, 95% CI 3.67–5.28), CKD (HR 1.68, 95% CI 1.41–2.01), all-cause mortality (HR 2.91, 95% CI 2.53–3.34), and CVD mortality (HR 3.75, 95% CI 2.93–4.80) [39]. More recent evidence extends this adverse phenotype to brain health: in 22,989 UK Biobank participants with T2D, 2843 developed AF during a median follow-up of 12.3 years, and new-onset AF was associated with higher risks of all-cause dementia (HR 2.15, 95% CI 1.80–2.57), Alzheimer’s disease (HR 1.44, 95% CI 1.06–1.96), and vascular dementia (HR 3.11, 95% CI 2.32–4.17) [40]. Concordantly, a 2024 clinical study of 160 patients—50 with T2D, 54 with T2D and AF, and 56 controls—showed significantly worse Mini-Mental State Examination, Montreal Cognitive Assessment, Activities of Daily Living, Instrumental Activities of Daily Living, and Geriatric Depression Scale scores in patients with combined T2D and AF, with each additional year of age increasing the risk of early cognitive decline by 7.3% in the T2D-AF group [41]. Collectively, these data indicate that the coexistence of diabetes and AF should not be viewed as simple comorbidity accumulation, but as an integrated cardiovascular–renal–cerebral risk state requiring active AF detection, structured symptom assessment, aggressive risk-factor modification, optimized anticoagulation when indicated, and longitudinal surveillance for HF, CKD, mortality, and cognitive decline.

The principal epidemiological studies examining the association between diabetes and AF, are summarized in Table 1.

## 4. Diabetic Atrial Cardiomyopathy as a Conceptual Framework: Mechanisms Linking Diabetes to Atrial Fibrillation

In this review, diabetic atrial cardiomyopathy is used descriptively as a unifying mechanistic framework, not as a formally established or uniform clinical diagnosis. Although experimental models can isolate hyperglycaemia, impaired insulin signalling, or related metabolic perturbations, human data are frequently confounded or mediated by obesity, hypertension, CKD, HF, OSA, ageing, and differences in treatment exposure. The pathways discussed below should therefore be interpreted as interacting components of a broader cardiometabolic atrial substrate; where possible, evidence supporting a diabetes-associated effect is distinguished from evidence reflecting cumulative risk-factor clustering.

The principal mechanisms linking diabetes to AF, the supporting evidence base, and their relevance to human disease are summarized in Table 2.

### 4.1. Fibrotic and Structural Remodelling

**Figure 1:**
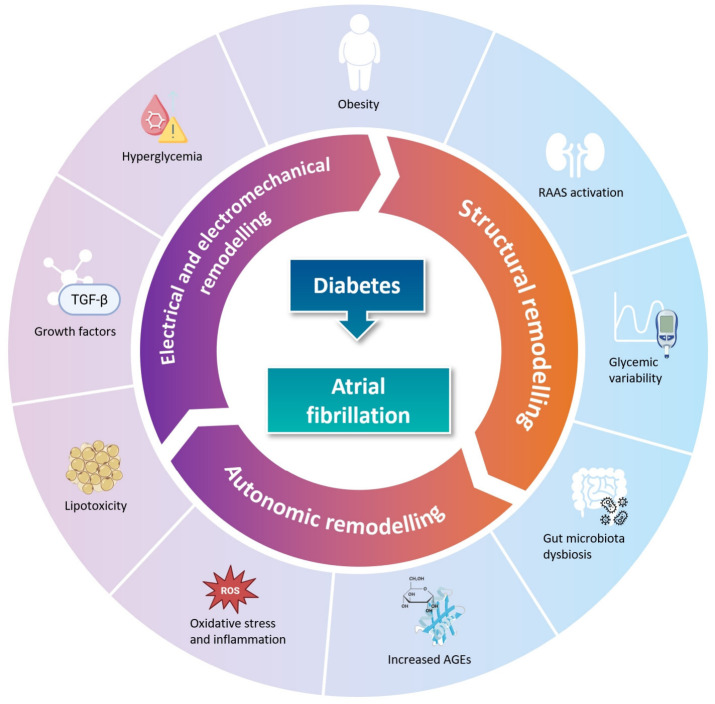
Mechanistic pathways linking diabetes to atrial fibrillation. Diabetes may promote atrial fibrillation through interrelated structural, electrical/electromechanical, and autonomic remodelling pathways. Hyperglycaemia, glycaemic variability, obesity, renin–angiotensin–aldosterone system activation, gut microbiota dysbiosis, advanced glycation end products, oxidative stress and inflammation, lipotoxicity, growth-factor signalling, and obstructive sleep apnoea converge to promote atrial fibrosis, conduction heterogeneity, autonomic imbalance, and substrate vulnerability for atrial fibrillation initiation and maintenance. Obstructive sleep apnoea may further amplify this substrate through intermittent hypoxaemia, negative intrathoracic pressure swings, autonomic fluctuations, atrial stretch, oxidative stress, and inflammatory activation.

Diabetes may contribute to AF through a convergent programme of atrial structural remodelling in which hyperglycaemia, advanced glycation end products, oxidative stress, inflammation, mitochondrial dysfunction, and fibroblast activation may progressively render the atrium a conductionally heterogeneous and re-entry-prone substrate [40,41,43] (Figure 1). A seminal experimental study by Kato et al. [43] showed that streptozotocin-induced diabetic rats developed marked hyperglycaemia and poor glycaemic control compared with controls (plasma glucose 34.5 ± 1.9 vs. 9.5 ± 0.1 mmol/L; haemoglobin A1c 10.0 ± 0.5% vs. 3.2 ± 0.1%), accompanied by a more than twofold increase in left atrial fibrotic deposition (32.7 ± 1.6% vs. 14.5 ± 1.1%); importantly, inhibition of advanced glycation end product formation with OPB-9195 reduced atrial fibrosis to 20.7 ± 2.4% despite persistent hyperglycaemia and attenuated the approximately threefold diabetes-induced upregulation of connective tissue growth factor, supporting the advanced glycation end product–receptor axis as a potential mediator of profibrotic atrial remodelling rather than a simple epiphenomenon of elevated glucose [43]. This aligns with the broader biology of diabetic cardiac fibrosis, in which activated fibroblasts, macrophages, cardiomyocytes, endothelial cells, transforming growth factor-β/Smad signalling, matrix cross-linking, endothelin-1, renin–angiotensin–aldosterone signalling, and reactive oxygen species may collectively increase extracellular matrix deposition and myocardial stiffness [44]. Oxidative stress may provide a particularly important mechanistic bridge between metabolic injury and atrial fibrosis: in diabetes, reactive oxygen species arise from mitochondrial electron leakage, nicotinamide adenine dinucleotide phosphate oxidase, xanthine oxidase, uncoupled nitric oxide synthase, protein kinase C activation, and advanced glycation end product–receptor signalling, with nuclear factor-κB activation amplifying inflammatory, hypertrophic, and profibrotic gene expression [45]. Human atrial tissue data support the translational relevance of this pathway: Anderson et al. studied right atrial appendage samples from 13 non-diabetic and 11 T2D patients undergoing coronary artery bypass grafting and found higher haemoglobin A1c (7.26 ± 2.49% vs. 5.78 ± 0.38%) and triglycerides (232.6 ± 46.7 vs. 164.7 ± 72.7 mg/dL) in T2D, approximately twofold greater atrial intramyocellular triglyceride content, reduced maximal fatty-acid-supported mitochondrial respiration, increased mitochondrial hydrogen peroxide emission, depleted glutathione, and biochemical evidence of persistent oxidative stress, suggesting that diabetic atria may be metabolically predisposed to redox-related structural and electrophysiological instability [46]. Recent primary studies further refine this model. In T2D db/db mice, Bohne et al. demonstrated high AF susceptibility with atrial electrical and structural remodelling, while glucagon-like peptide-1 and liraglutide reduced AF and prevented atrial remodelling, supporting the concept that metabolic therapies may modify the arrhythmogenic substrate rather than only systemic risk [47]. In a 2024 study [48], high-fat diet plus low-dose streptozotocin induced severe hyperglycaemia, atrial fibrosis, calpain activation, calcium-handling dysfunction, and AF susceptibility; calpastatin overexpression prevented diabetes-induced fibrosis and AF vulnerability despite comparable hyperglycaemia, supporting calpain-mediated disruption of calcium homeostasis and structural integrity as a potential mechanistic link between diabetes and atrial remodelling. Complementary evidence from Zhou et al. [49] showed that high-fat diet diabetic mice had higher fasting glucose (168 ± 16 vs. 119 ± 11 mg/dL), greater body weight (57 ± 1 vs. 35 ± 2 g), worse diastolic function (E/e′ 23.2 ± 1.7 vs. 13.6 ± 1.0), and markedly greater AF inducibility (6/6 vs. 1/6 mice), but without increased atrial collagen I, suggesting that inflammatory–redox–calcium leak mechanisms may precede overt fibrosis; diabetes increased atrial interleukin-1β (2.83 ± 0.74 vs. 0.91 ± 0.07), doubled mitochondrial reactive oxygen species (266 ± 14% vs. 100 ± 12%), and increased oxidized CaMKII and RyR2 phosphorylation, whereas macrophage depletion, interleukin-1β neutralization, mitoTEMPO, and RyR2 stabilization each reduced AF vulnerability. Finally, the 2024 study by Peng et al. [52] provides a mitochondrial-transcriptional perspective, suggesting that NR4A3 may mitigate diabetes-associated atrial cardiomyopathy through preservation of succinate dehydrogenase subunit A expression, improved mitochondrial energy metabolism, and reduced oxidative injury.

### 4.2. Diabetes-Driven Atrial Electrical and Electromechanical Remodelling

Diabetes may contribute to AF not only through a fibrotic atrial substrate, but also through electrical and electromechanical abnormalities that may impair impulse propagation, increase repolarization heterogeneity, and weaken atrial mechanical synchrony. Clinically, these changes are detectable before overt arrhythmia: in a tissue Doppler and surface electrocardiography study of 88 patients with T2D and 49 matched controls, maximum P-wave duration and P-wave dispersion were significantly higher in T2D (105.7 ± 10.2 vs. 102.2 ± 7.5 ms; 40.6 ± 7.6 vs. 33.6 ± 5.9 ms), while interatrial, intra-atrial, and intra-left atrial electromechanical delay were also prolonged (16.5 ± 7.8 vs. 11.2 ± 4.4 ms; 9.0 ± 7.3 vs. 6.0 ± 3.8 ms; 7.4 ± 5.2 vs. 5.1 ± 3.2 ms, respectively), with interatrial delay correlating with both P-wave dispersion and left atrial volume, thereby linking electrical inhomogeneity to atrial structural loading [53]. Similar abnormalities appear early along the glycaemic continuum: in patients with impaired fasting glucose, interatrial and intra-atrial electromechanical delays were approximately doubled compared with controls (34.0 [17.0] vs. 17.0 [4.0] ms and 15.0 [8.5] vs. 7.5 [2.0] ms), and glucose levels were strongly associated with interatrial delay (r = 0.76; β = 0.753), suggesting that atrial electromechanical uncoupling may begin before established diabetes [54]. Experimental models provide mechanistic depth to these clinical observations. In alloxan-induced diabetic rabbits, atrial effective refractory period dispersion and interatrial conduction time were increased, atrial electromechanical indices correlated with fibrosis burden, and AF inducibility rose from 1/8 controls to 6/8 diabetic animals [55]. Optical mapping in streptozotocin-induced diabetic rats further demonstrated longer atrial tachyarrhythmia duration (2.4 ± 0.6 vs. 0.9 ± 0.3 s), slower and more heterogeneous conduction, prolonged action potential duration at 80% repolarization (53 ± 2 vs. 40 ± 3 ms), greater spatial dispersion, and universal action potential duration alternans (100% vs. 0%), accompanied by increased interstitial fibrosis and reduced connexin-40 expression [56]. At the ionic level, diabetes is associated with remodelling of both depolarizing and repolarizing currents: streptozotocin-induced diabetic mice showed 85% and 92% downregulation of atrial small-conductance calcium-activated potassium channel 2 and 3 proteins, respectively, reduced small-conductance calcium-activated potassium current, action-potential prolongation, increased oxidative stress, and accelerated channel turnover, while subsequent work linked small-conductance calcium-activated potassium channel 2 loss to F-box protein-32-dependent ubiquitin–proteasome degradation [57,58]. In parallel, increased late sodium current in diabetic mice prolonged action potential duration at 50% and 90% repolarization, increased AF susceptibility, and was attenuated by the late sodium current inhibitor GS967 [59]. Loss of insulin signalling may also influence atrial excitability: in type 1 diabetic Akita mice, AF susceptibility was accompanied by prolonged P-wave duration, slowed atrial conduction, reduced sodium current and Kv1.5-mediated potassium current, lower SCN5A/Nav1.5 expression, and prolonged action potential duration, whereas chronic insulin shortened P-wave duration, improved conduction, increased sodium current, and reduced AF susceptibility and duration [60]. These findings are reinforced by contemporary metabolic-signalling studies: atrium-selective AMPK deletion produced early ectopy, downregulation of connexins and ion-channel subunits, a 50% reduction in left atrial upstroke velocity, a 40% increase in left atrial activation time, and spontaneous AF in 50% and 90% of mice by 3 and 6 months, respectively, before overt left atrial fibrosis became dominant [50]. Recent primary studies provide further mechanistic support, showing that T2D db/db mice exhibit impaired atrial conduction and combined electrical–structural remodelling, that oxidized calcium/calmodulin-dependent protein kinase II and O-GlcNAcylation are associated with greater AF susceptibility through distinct diabetic mechanisms, and that downstream inflammatory–redox–calcium pathways—including macrophage interleukin-1β signalling, calpain activation, and Zbtb16–Txnip–Trx2 signalling—may promote abnormal calcium release, mitochondrial oxidative stress, conduction vulnerability, and AF inducibility [49,61,62]. Importantly, the reversibility of this substrate is increasingly plausible: glucagon-like peptide-1 and liraglutide reduced AF and prevented atrial remodelling in T2D db/db mice, supporting the hypothesis that diabetes-related AF may reflect an active metabolic-electrophysiological disease process rather than irreversible electrical scarring alone [47].

### 4.3. Autonomic Remodelling

Diabetes may contribute to AF through autonomic remodelling that extends beyond generalized cardiovascular autonomic neuropathy (CAN) to include sympathovagal imbalance, impaired parasympathetic responsiveness, heterogeneous sympathetic innervation, and remodelling of intrinsic cardiac ganglia. CAN is a frequent and clinically important diabetic complication, often evolving from subclinical abnormalities in baroreflex sensitivity and HRV to overt resting tachycardia, exercise intolerance, orthostatic hypotension, and excess mortality; in the DCCT, intensive glycaemic control reduced CAN incidence by approximately 50% over 6.5 years, whereas multifactorial intervention in Steno-2 reduced progression to diabetic autonomic neuropathy from 65% to 49%, emphasizing that autonomic injury is modifiable but closely linked to cumulative metabolic and vascular stress [63]. A meta-analysis of 25 case–control studies including 2932 participants confirmed a broad depression of autonomic modulation in T2D, with significantly lower RR intervals, standard deviation of normal-to-normal intervals, root mean square of successive differences, pNN50, total power, low-frequency power, and high-frequency power, indicating impairment of both sympathetic and parasympathetic components rather than an isolated vagal defect [64]. The clinical link with AF is supported by Rizzo et al., who screened 1992 patients with T2D using repeated 48 h Holter monitoring at 3, 6, 9, and 12 months; among selected patients, 176 had silent AF episodes and 288 did not, and the silent AF group showed a significantly higher low-frequency/high-frequency ratio throughout monitoring, with absolute AF burden correlating positively with this ratio (r = 0.31, *p* < 0.001), while multivariable analysis identified the low-frequency/high-frequency ratio as an independent determinant of AF episodes [65]. Mechanistically, autonomic dysregulation may lower atrial refractoriness, increase spatial dispersion, facilitate triggered activity, and interact with structural remodelling; in a broad mechanistic synthesis of AF, autonomic neural dysregulation was recognized as one of the four core disturbance domains promoting ectopic firing and re-entry, and diabetes was specifically linked to both structural and autonomic remodelling [66]. Experimental studies provide mechanistic support for this association. In streptozotocin-induced diabetic rats, sympathetic nerve stimulation increased AF incidence from 14 ± 6% to 30 ± 8% (*p* < 0.01), but not in controls, and this was accompanied by increased atrial effective refractory period heterogeneity and heterogeneous tyrosine hydroxylase-positive sympathetic innervation; parasympathetic stimulation also increased AF incidence in both control and diabetic hearts, underscoring the arrhythmogenic potential of abrupt autonomic shifts [67]. In Akita diabetic mice, carbachol decreased heart rate by only 12% versus 30% in wild-type mice and increased corrected sinoatrial node recovery time by only 37% versus 123%, reflecting blunted parasympathetic responsiveness due to reduced acetylcholine-activated potassium current and impaired insulin-dependent phosphoinositide 3-kinase signalling [68]. Additional autonomic-anatomic evidence comes from diabetic pulmonary vein ganglia: in Akita mice, both sympathetic and parasympathetic neuronal somas were hypotrophied, pulmonary vein ganglion stimulation produced less P–P interval prolongation, atropine caused less P–P interval shortening, and modelling suggested reduced sympathetic and parasympathetic ganglionic activity, supporting a potential role for diabetes-associated intrinsic cardiac neural remodelling at a key pulmonary vein trigger region [69]. More recent population data reinforce the clinical relevance of this autonomic phenotype: in the Fremantle Diabetes Study Phase II [70], among 830 community-based participants with T2D and valid cardiovascular autonomic reflex tests, 33.7% had possible CAN and 15.3% had definite CAN, and definite CAN was associated with a graded increase in mortality, with possible CAN and definite CAN conferring adjusted all-cause mortality hazard ratios of 1.47 and 2.42, respectively [70].

### 4.4. Glycaemic Fluctuations as an Arrhythmogenic Metabolic Stressor in Diabetes

Mean HbA1c and glycaemic variability describe different aspects of glucose exposure. HbA1c approximates average glycaemia over preceding months, whereas within-day or visit-to-visit variability reflects the amplitude and frequency of glucose fluctuations; neither measure should be regarded as a surrogate for diabetes duration or diabetes-related organ damage.

Glycaemic variability has emerged as a mechanistically important dimension of diabetes-related AF risk, distinct from mean glycaemic exposure alone. Biological plausibility is supported by Monnier et al. [71], who showed in 21 patients with T2D and 21 matched controls that oxidative stress, assessed by urinary 8-iso prostaglandin F2α, was higher in T2D than in controls (482 ± 206 vs. 275 ± 85 pg/mg creatinine), and correlated more strongly with mean amplitude of glycaemic excursions than with conventional indices of chronic hyperglycaemia (r = 0.86, *p* < 0.001; standardized β = 0.830), supporting the hypothesis that acute glucose swings may act as a potent redox stimulus [71]. Experimental data support a link between this metabolic volatility and AF substrate formation: in streptozotocin-induced diabetic rats, Saito et al. modelled glucose fluctuations through repeated 24 h fasting and insulin-induced glucose lowering three times weekly for 3 weeks; compared with controlled and persistently uncontrolled diabetes, fluctuating glycaemia produced the greatest atrial fibrosis, higher collagen type 1, collagen type 3, α-smooth muscle actin, malondialdehyde, thioredoxin-interacting protein, caspase-3 expression, and TUNEL-positive cell burden, with the highest AF inducibility, suggesting that repeated oscillations between hypo- and hyperglycaemia may amplify oxidative, apoptotic, and fibrotic remodelling beyond sustained hyperglycaemia alone [72]. This concept helps reconcile why intensive glucose lowering does not necessarily reduce AF: in the ACCORD analysis of 10,082 patients with T2D randomized to intensive HbA1c targeting below 6.0% versus standard targeting of 7.0–7.9%, incident AF occurred in 159 patients (1.58%), with no significant difference between intensive and standard therapy (5.9 vs. 6.37 events per 1000 patient-years; *p* = 0.52), while new-onset AF identified a markedly adverse phenotype associated with all-cause mortality (hazard ratio 2.65), myocardial infarction (hazard ratio 2.10), and HF (hazard ratio 3.80) [42]. Conversely, long-term HbA1c instability appears clinically informative: in 505 patients with T2D followed for a median of 6.9 years, Gu et al. reported 48 incident AF cases (9.5%), and both HbA1c standard deviation (hazard ratio 1.726, *p* = 0.001) and HbA1c coefficient of variation (hazard ratio 1.241, *p* = 0.024) independently predicted new-onset AF after adjustment for age, body mass index, left ventricular mass index, and left atrial diameter, with proposed thresholds of 0.665% for HbA1c standard deviation and 8.97% for HbA1c coefficient of variation [73]. In a larger Korean nationwide cohort of 1,509,280 adults with T2D [74], severe hypoglycaemia affected 10,864 individuals (0.72%) before baseline assessment, and 48,916 first-time AF events (3.24%) occurred over 8.5 years; prior severe hypoglycaemia independently predicted new-onset AF (hazard ratio 1.10, 95% confidence interval 1.01–1.19), potentially through adrenergic activation, repolarization instability, autonomic dysfunction, and pro-inflammatory/prothrombotic stress [74]. More recent primary data extend these observations: in 27,246 adults with T2D followed for a median of 70.7 months, Hsu et al. [75] found an AF incidence of 21.31 per 1000 person-years, with the highest HbA1c variability score independently associated with new-onset AF (hazard ratio 1.29, 95% confidence interval 1.12–1.50), although fasting-glucose coefficient of variation was not independently associated with AF after full adjustment. In the perioperative setting, Zhou et al. [95] analysed 5365 cardiac-surgery patients from MIMIC-IV and found that 1056 (19.7%) developed postoperative AF; compared with low glycaemic variability, moderate and high variability were associated with substantially higher postoperative AF risk (odds ratios 1.82 and 2.25, respectively), with glycaemic variability showing good discrimination for postoperative AF (area under the curve 0.77) [95].

Clinical translation remains preliminary. The 2026 American Diabetes Association Standards of Care recognize continuous glucose monitoring (CGM)-derived time in range (TIR), time below range, time above range, and glucose coefficient of variation as complementary measures of glycaemic control and recommend CGM according to individual diabetes-management requirements, particularly for people using insulin or at risk of hypoglycaemia [96,97]. Neither the 2024 ESC AF guideline nor current diabetes guidance, however, recommends CGM metrics specifically for AF screening, risk stratification, or rhythm-control decisions. In patients with AF, these indices should therefore be used to optimize diabetes care and identify clinically relevant hypo- or hyperglycaemic excursions rather than as validated arrhythmia biomarkers. Randomized and post hoc evidence suggests that SGLT2 inhibitors and GLP-1 receptor agonists can improve TIR, postprandial glucose excursions, or other measures of glycaemic variability without a commensurate increase in hypoglycaemia [76,98]. Whether attenuation of glycaemic variability independently reduces AF incidence, burden, or recurrence has not been established and remains hypothesis-generating.

### 4.5. Obesity, Insulin Resistance, and Adipose–Atrial Crosstalk in Diabetic Atrial Cardiomyopathy

Obesity and insulin resistance (IR) may increase AF susceptibility through the convergence of systemic metabolic stress with local atrial adiposity, haemodynamic loading, inflammation, oxidative stress, gut-derived endotoxaemia, and ion-channel remodelling. Genetic evidence supports a potentially causal contribution of adiposity, with a two-sample Mendelian randomization study showing that each genetically determined 1-SD increment in BMI increased AF risk by 42.5% (odds ratio 1.43), while IR appears to add a partly independent metabolic signal: in 11,851 ARIC participants without known CVD, the triglyceride–glucose index showed a U-shaped association with incident AF over 24.26 years, with 1925 AF cases and higher risk at both low and high index values compared with the reference range of 8.80–9.20 (adjusted hazard ratio 1.15 for <8.80 and 1.18 for >9.20) [77,78]. Mechanistically, obesity may act not only as an anthropometric exposure but also as a modifier of the atrial substrate. Human EAT, which is anatomically contiguous with the myocardium, has a distinct transcriptomic profile: in 41 surgical patients, 2728 genes were upregulated in EAT compared with subcutaneous adipose tissue, with 400 common EAT-enriched genes related to extracellular matrix remodelling, inflammation, infection, and thrombosis; notably, periatrial EAT showed enrichment for pathways involved in cardiac muscle contraction and intracellular calcium signalling, supporting a location-specific interaction with atrial electrophysiology [79]. Venteclef et al. provided functional evidence for this paracrine axis by studying paired EAT and subcutaneous adipose tissue samples from 39 patients undergoing coronary bypass surgery; EAT secretome, but not subcutaneous adipose tissue secretome, induced interstitial and peripheral fibrosis in rat atrial organ culture, with Activin A highly expressed in EAT and neutralizing antibody attenuating its profibrotic effect [80]. This adipose–atrial cross-talk extends beyond soluble mediators: in 32 patients with AF and 30 without AF, EAT-derived extracellular vesicles from AF patients carried a distinctive proinflammatory, profibrotic, and proarrhythmic signature, stimulated migration and proliferation of stromal and endothelial cells, shortened action potential duration, and uniquely induced sustained rotor-like re-entry in human induced pluripotent stem cell-derived cardiomyocyte sheets [81]. More recent primary data refine this extracellular-vesicle mechanism, showing that EAT-derived vesicles contain 824 detected microRNAs and that miR-1-3p and miR-133a-3p were enriched in EAT compared with subcutaneous adipose tissue; overexpression of these candidates slowed conduction and reduced Kcnj2 and Kcnj12 expression, supporting a potential role for adipose-derived microRNA cargo in obesity-related atrial conduction vulnerability [82]. Experimental obesity models further suggest that the arrhythmogenic substrate is multimodal: in Zucker obese rats exposed to simulated obstructive sleep apnea, AF was induced in 24 of 28 obese rats (85.7%) versus 5 of 18 lean rats (27.8%), whereas inferior vena cava occlusion preventing acute left atrial distension reduced AF by 83.3%, supporting an interaction between obesity, diastolic dysfunction, sleep apnea, and atrial stretch [83]. In diet-induced obese mice, pacing-induced AF occurred in 100% versus 25% of controls and was accompanied by reduced Nav1.5 expression, lower sodium and L-type calcium currents, increased Kv1.5/ultra-rapid potassium current, shortened action potential duration, increased NOX2/protein kinase C signalling, oxidative stress, and atrial fibrosis; mitochondrial antioxidant therapy reversed both ion-channel and structural remodelling, suggesting that redox stress may represent a modifiable mechanistic node [51]. Preclinical evidence further supports a mechanistic, rather than merely epiphenomenal, role for obesity-related inflammatory remodelling: NLRP3 inflammasome activation increased in atrial tissue from obese patients, obese sheep, and high-fat-diet mice, while NLRP3 deletion protected high-fat-diet mice from AF, prevented Kv1.5 upregulation, attenuated profibrotic signalling, and reduced abnormal sarcoplasmic-reticulum calcium release [84]. The gut–heart axis may add another layer to obesity-related AF: transplantation of high-fat-diet microbiota into normally fed mice increased Desulfovibrionaceae, circulating lipopolysaccharide, left atrial inflammatory cytokines, ferroptosis, and Toll-like receptor 4/nuclear factor-κB/NLRP3 signalling, whereas inhibition of ferroptosis or NLRP3 attenuated atrial fibrosis and AF susceptibility [85]. Importantly, weight loss may partially reverse this atrial vulnerability: in 37 bariatric-surgery patients without AF, BMI fell from 43.2 ± 5.2 to 28.9 ± 4.6 kg/m^2^, EAT volume from 132 ± 49 to 87 ± 52 mL, and P-wave duration from 109 ± 11 to 102 ± 11 ms at 1 year, although the reduction in P-wave duration was not directly explained by changes in EAT volume or BMI alone [86].

### 4.6. Lipotoxicity and Metabolic Substrate Remodelling

Lipotoxicity provides a mechanistic bridge between obesity, insulin resistance, T2D, and AF by linking excess fatty-acid delivery to mitochondrial overload, impaired fatty-acid oxidation (FAO), accumulation of toxic lipid intermediates, oxidative stress, inflammation, apoptosis, gap-junction disturbance, fibrosis, and conduction instability [87]. Human metabolic-imaging data support the concept that lipid overload begins early in the diabetic continuum: in a ^1^H-magnetic resonance spectroscopy study of 134 individuals, myocardial triglyceride content was already 2.3-fold higher in impaired glucose tolerance and 2.1-fold higher in T2D compared with lean normoglycaemic controls, despite preserved LV ejection fraction, indicating that cardiac steatosis can precede overt systolic dysfunction [88]. Consistently, human atrial tissue from patients with T2D shows increased intramyocellular triglyceride content, impaired maximal mitochondrial oxidation of palmitoyl-carnitine and glutamate, and increased mitochondrial oxidant emission, suggesting that the diabetic atrium is exposed simultaneously to lipid oversupply and reduced oxidative reserve [46]. Foundational experimental work suggests that excess cardiomyocyte lipid uptake may be pathogenic: cardiomyocyte-surface lipoprotein lipase increased myocardial lipid uptake, induced myocyte enlargement and abnormal architecture, and produced dilated cardiomyopathy, supporting a potentially causal role of lipid excess in myocardial remodelling [89]. The arrhythmogenic potential of lipid intermediates is also illustrated by inherited FAO disorders, in which 24 of 107 children presented predominantly with arrhythmias or conduction defects, including 15 ventricular tachycardias, 4 atrial tachycardias, 4 sinus-node dysfunction cases with atrial tachycardia, 6 atrioventricular blocks, and 4 left bundle-branch blocks; mechanistically, long-chain acylcarnitines were proposed to disrupt sarcolemmal phospholipids, depress sodium and inward-rectifier potassium currents, activate calcium currents, and impair gap junctions [90]. In AF itself, lipid remodelling may become self-perpetuating: irregular pacing increased diastolic calcium, activated CaMKII and AMPK, increased FAT/CD36 membrane expression and palmitate uptake, reduced GLUT4-mediated glucose uptake, and produced lipid accumulation in cardiomyocytes, with similar changes observed in spontaneous AF mice and human left atrial myocardium from AF patients [91]. The most direct obesity-related AF evidence comes from Zhang et al., who fed mice an HFD and treated them with LCA at 150 mg/kg/day; HFD increased AF vulnerability and produced LA dilatation, cardiomyocyte hypertrophy, Cx43 remodelling, fibrosis, defective cardiac FAO, lipid accumulation, oxidative stress, DNA damage, inflammation, and insulin resistance, whereas LCA restored FAO through AMPK/PGC1α activation, attenuated atrial lipotoxicity, reduced structural remodelling, and ameliorated AF susceptibility [92]. This FAO–AMPK axis is reinforced by atrium-selective AMPK deletion, which caused spontaneous AF in 50% and 90% of mice by 3 and 6 months, respectively, preceded by ectopy, reduced connexin and Nav1.5 expression, a 50% decrease in LA upstroke velocity, and a 40% increase in LA activation time, suggesting that impaired metabolic sensing may destabilize atrial excitability before advanced structural remodelling becomes dominant [50]. More recent primary evidence provides further mechanistic support: a 2024 ANXA1–FPR2 study identified FPR2 as an obesity-related AF hub gene and showed that Ac2-26 reduced AF susceptibility in HFD-induced obese mice by attenuating atrial fibrosis, lipid deposition, oxidative injury, and apoptosis, whereas myocardial AMPK knockdown reversed these protective effects; in vitro, Ac2-26 also reduced palmitate-induced lipid deposition, oxidative injury, and apoptosis through AMPK-dependent enhancement of FA catabolism [99].

## 5. Atrial Fibrillation Classification and the AF-CARE Framework in Diabetes

**Figure 2:**
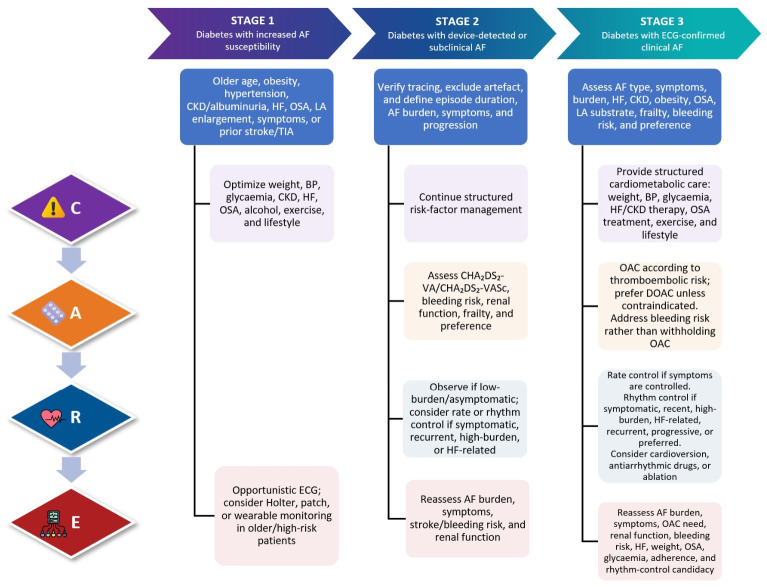
Stage-specific AF-CARE framework for diabetes-associated atrial fibrillation. The figure summarizes a staged approach to AF management in diabetes, progressing from individuals with increased AF susceptibility, to device-detected or subclinical AF, and finally to electrocardiogram-confirmed clinical AF. Across stages, management is structured according to the AF-CARE pathway: [C] comorbidity and risk-factor management, [A] avoidance of stroke and thromboembolism, [R] symptom reduction through rate or rhythm control, and [E] evaluation with dynamic reassessment. The framework highlights how priorities shift from early cardiometabolic optimization and rhythm screening, to AF-burden confirmation and individualized anticoagulation decisions, and ultimately to comprehensive management of clinical AF with stroke prevention, symptom control, and longitudinal reassessment.

For integrated management, AF in diabetes can be considered across three clinically relevant stages: individuals at risk for AF, defined as those without documented AF but with diabetes and often additional cardiometabolic risk factors; device-detected subclinical AF, referring to asymptomatic atrial arrhythmia episodes identified by implanted or wearable monitors without prior surface ECG documentation; and clinical AF, confirmed by ECG or rhythm strip [9,100]. This staging aligns with the contemporary AF-CARE pathway [9]—[C] comorbidity and risk-factor management, [A] avoid stroke and thromboembolism, [R] reduce symptoms by rate and rhythm control, and [E] evaluation and dynamic reassessment—and provides a practical structure for tailoring management priorities across the diabetic AF continuum (Figure 2).

Throughout the following management sections, guideline-based recommendations are distinguished from randomized clinical evidence and from observational or hypothesis-generating findings.

The principal therapeutic strategies for AF in diabetes are summarized in Table 3, highlighting their practical application, main clinical rationale, and key limitations across prevention, stroke reduction, rhythm management, and longitudinal reassessment.

### 5.1. Diabetes and Increased Risk for Atrial Fibrillation

Individuals with diabetes at risk for AF should be conceptualized as a heterogeneous cardiometabolic population in whom dysglycaemia may interact with obesity, hypertension, albuminuria, CKD, HF, obstructive sleep apnea (OSA), lifestyle exposures, and microvascular disease to accelerate atrial substrate formation. In the Swedish National Diabetes Register [101], including 83,162 patients with T2D and no baseline AF followed for a mean of 6.8 years, updated BMI, obesity, updated SBP, hypertension, and cumulative microalbuminuria were independently associated with incident AF, with HRs of 1.31 per 5 kg/m^2^, 1.51 for obesity, 1.13 per 10 mmHg SBP, 1.71 for hypertension, and 1.21 for microalbuminuria; HF was an especially strong determinant, with HRs of 1.76 for prior HF and 2.56 for in-study HF [101]. Microvascular disease further refines risk: in ACCORD, among 7603 adults with T2D and no baseline AF, 63.3% had diabetic kidney disease, retinopathy, or neuropathy, and microvascular disease was associated with a 1.88-fold higher risk of incident AF, increasing from RR 1.62 with one affected vascular bed to RR 2.47 with at least two affected beds [93]. OSA should also be actively sought because, in a matched UK cohort of patients with T2D, incident OSA was associated with higher risks of composite CVD, HF, stroke/TIA, CKD, mortality, and AF specifically (adjusted HR 1.53, 95% CI 1.28–1.83) [94]. Mechanistically, intermittent hypoxaemia, autonomic fluctuations, negative intrathoracic pressure swings, oxidative stress, sarcoplasmic-reticulum Ca^2+^ leak, increased late sodium current, and reduced connexin-43 expression may amplify the fibrotic, electrical, and autonomic remodelling already associated with diabetic atrial cardiomyopathy [102]. OSA is also frequently under-recognized in women, who may present with fatigue, insomnia, headache, mood disturbance, or impaired concentration rather than classical symptoms, contributing to delayed diagnosis and undertreatment [103].

These observations also have implications for sex-sensitive risk assessment. Although men generally have higher absolute AF incidence, the relative increment associated with diabetes may be greater in women, particularly at younger and middle ages [104]. Sex-stratified analyses from ACCORD and NOMED-AF remain limited and do not support different screening thresholds; however, atypical symptoms and the under-recognition of AF and OSA in women argue against restricting rhythm surveillance to patients with classical presentations.

Lifestyle factors are similarly actionable: in 2,551,036 Korean patients with T2D, ex-smoking and current smoking were associated with modestly higher AF risk, moderate and heavy alcohol intake conferred HRs of 1.12 and 1.24, respectively, whereas moderate-to-vigorous physical activity was associated with lower AF risk (HR 0.93); a 2024 nationwide study of 2,392,486 patients further showed that physical activity was associated with lower AF risk across diabetes-duration strata, with 1000–1499 MET-min/week associated with the lowest overall AF risk and ≥1500 MET-min/week showing the greatest relative risk reduction in diabetes duration ≥10 years [105,106]. Pharmacological cardiometabolic optimization may add benefit: SGLT2 inhibitors reduced AF/AFL in a meta-analysis of 16 RCTs including 38,335 patients with T2D (RR 0.76, 95% CI 0.65–0.90), and real-world Taiwanese data showed lower new-onset AF risk with SGLT2 inhibitors versus DPP4 inhibitors (HR 0.61, 95% CI 0.50–0.73) [107,108]. The pharmacological findings should be interpreted according to study design: the pooled randomized evidence provides stronger support than the real-world comparison, although AF was not uniformly a prespecified primary outcome and the available data do not establish an AF-specific treatment indication.

Evaluation should move beyond a single baseline estimate of AF risk toward repeated rhythm surveillance and iterative reassessment of comorbidity control, thromboembolic risk, treatment adherence, and symptom burden. The 2023 ESC diabetes guideline [109] notes that silent AF is not uncommon in diabetes and recommends opportunistic AF screening by pulse palpation or ECG, with repeated surface ECG, Holter ECG, patient-activated recording, or wearable devices considered in symptomatic or high-risk individuals without definite AF. The 2024 ESC AF guideline [9] formalizes this approach within AF-CARE, including “[C] comorbidity and risk factor management” and “[E] evaluation and dynamic reassessment”, and supports population-based prolonged non-invasive ECG screening in individuals aged ≥75 years or ≥65 years with additional CHA_2_DS_2_-VA risk factors; it also recommends normal weight, active lifestyle, avoidance of alcohol excess, weight reduction in obesity, and consideration of metformin or SGLT2 inhibitors when pharmacological diabetes treatment is needed to prevent AF. Randomized screening evidence supports the diagnostic yield of structured approaches: a network meta-analysis of 9 RCTs including >85,000 older adults found that AF screening increased new AF detection and OAC initiation, with the clearest benefit driven by systematic rather than opportunistic screening, although mortality and stroke reductions were not statistically definitive [110]. In STROKESTOP, which randomized 27,975 individuals aged 75/76 years, modelled screening effects over 6.9 years suggested that, per 1000 individuals invited, screening gained 77 life-years and 65 QALYs while reducing lifetime costs by €1.77 million [111]. Dynamic reassessment is particularly relevant in diabetes because age, hypertension, HF, CKD, albuminuria, obesity, and vascular disease evolve over time; in the 2025 Norwegian AFNOR analysis, CHA_2_DS_2_-VA score increased in 50% of initially low- or intermediate-risk AF patients after a median 1.7 years, with increases already present in 19% at 6 months, 25% at 1 year, and 40% at 3 years, supporting systematic reassessment rather than static risk classification [112]. Accordingly, opportunistic or prolonged rhythm surveillance in selected high-risk individuals reflects guideline-based practice, whereas randomized evidence has established improved AF detection and greater OAC initiation but has not yet demonstrated a definitive reduction in stroke or mortality. The consideration of metformin or SGLT2 inhibitors is likewise guideline based when glucose-lowering therapy is otherwise indicated and should not be interpreted as definitive randomized evidence for prescribing either agent solely to prevent AF.

### 5.2. Diabetes and Subclinical Atrial Fibrillation

In patients with diabetes, subclinical AF should be interpreted as a signal of advanced cardiometabolic and atrial vulnerability rather than as an isolated rhythm abnormality [113]. Detection is strongly dependent on monitoring intensity: in a high-risk outpatient diabetes cohort, 7-day external loop recording identified silent AF or flutter in 10.5% of 200 patients, whereas standard resting electrocardiography detected none; age, male sex, and albuminuria were independently associated with silent AF [114]. Similarly, in patients aged ≥65 years with both hypertension and diabetes, insertable cardiac monitoring detected subclinical AF in 20.7% over a median of 588 days, compared with only 2.4% by 72 h Holter monitoring [115]. These observations support a structured AF-CARE approach in which comorbidity and risk-factor management is central: blood pressure control, effective glycaemic management, weight reduction in obesity [116], treatment of HF and CKD, evaluation for obstructive sleep apnoea, alcohol reduction, tailored exercise, and aggressive management of other modifiable risk factors should be integrated into the care pathway. The 2024 ESC AF guideline explicitly places these measures under “[C] Comorbidity and risk factor management”, recommends OAC according to thromboembolic risk rather than AF temporal pattern, and emphasizes reassessment of symptoms, stroke risk, bleeding risk, comorbidities, and AF burden at 6 months and at least annually thereafter [9]. The monitoring studies cited above are observational and primarily inform diagnostic yield; the recommendations regarding comorbidity management and longitudinal reassessment derive from the 2024 ESC AF guideline. Sex-specific evidence in device-detected AF remains limited. Male sex predicted silent AF in one high-risk diabetes cohort [114], but this observation should not be used to lower vigilance in women, in whom atypical symptoms and delayed diagnosis may reduce clinical detection.

Stroke prevention in diabetic patients with subclinical AF requires careful integration of arrhythmia burden with clinical risk. Observational device studies show a graded relation between AF duration and thromboembolism: in ASSERT, episodes >24 h were associated with stroke or systemic embolism (adjusted HR 3.24), whereas episodes between 6 min and 24 h were not significantly different from no subclinical AF; in TRENDS, a daily atrial tachyarrhythmia/AF burden ≥ 5.5 h was associated with an annualized thromboembolic risk of 2.4% versus 1.1% with no burden; and in SOS AF, a 1 h daily burden threshold carried the strongest association with ischemic stroke (HR 2.11) [117,118,119,120]. Randomized evidence now supports a more nuanced position: in ARTESIA, among 4012 patients with subclinical AF lasting 6 min to 24 h, apixaban reduced stroke or systemic embolism compared with aspirin (0.78% vs. 1.24% per patient-year; HR 0.63), but increased major bleeding (1.71% vs. 0.94% per patient-year; HR 1.80) [121]. By contrast, NOAH-AFNET 6 did not demonstrate a favourable net clinical benefit of edoxaban in patients with atrial high-rate episodes lasting ≥6 min and showed a higher incidence of the composite of death or major bleeding [122]. No validated minimum AF-burden threshold currently mandates OAC. As a pragmatic approach, in a patient with diabetes and a CHA_2_DS_2_-VA score ≥ 2, an adjudicated episode lasting <24 h should prompt individualized shared decision-making rather than automatic anticoagulation, whereas episodes lasting ≥24 h or progression to ECG-confirmed clinical AF strengthen the rationale for OAC when bleeding risk is acceptable [9,116,120,122]. Beyond episode duration, the decision should account for prior stroke or transient ischemic attack, renal function, albuminuria, frailty, bleeding susceptibility, and patient preference; when OAC is selected, DOACs are preferred except in patients with mechanical valves or moderate-to-severe mitral stenosis [9]. Thus, device-derived burden thresholds remain based largely on observational cohorts, ARTESIA provides randomized evidence regarding the trade-off between thromboembolic prevention and major bleeding, and the final treatment decision remains anchored to guideline-directed individualized assessment.

Rate and rhythm control are usually secondary considerations in subclinical AF because most episodes are asymptomatic and of low burden. In the LOOP natural-history analysis, AF ≥ 6 min was detected in 35% of high-risk individuals, but the median AF burden was only 0.13% of monitored time, symptoms were absent in 90% at AF debut, 87% never reported AF-related symptoms, and progression to 24 h episodes occurred in only 16% [123]. Thus, symptom-directed rate or rhythm control should be reserved for patients who develop clinical AF, rapid ventricular rates, HF deterioration, increasing AF burden, or impaired quality of life, while the dominant management priorities remain substrate control, stroke prevention, and longitudinal surveillance. Emerging digital and AI-enabled tools may refine this strategy: an AI-enabled sinus-rhythm electrocardiogram identified occult AF with an area under the curve of 0.87 in >180,000 patients, and AI-derived electrocardiographic ageing was associated with higher new-onset AF risk across multinational cohorts [124,125,126,127]. Overall, patients with diabetes with subclinical AF require dynamic phenotyping rather than a binary “treat or ignore” approach, with repeated reassessment of AF burden, metabolic control, renal status, bleeding risk, and net clinical benefit. AI-enabled ECG analysis and wearable-derived digital biomarkers remain investigational and should not be regarded as substitutes for validated, guideline-directed rhythm assessment.

### 5.3. Diabetes and Clinical Atrial Fibrillation

In diabetic patients with established clinical AF, management should be organized around substrate modification, cardiorenal protection, stroke prevention, symptom control, and repeated reassessment rather than around rhythm suppression alone. Registry data illustrate the adverse phenotype: in the EORP-AF General Pilot Registry, diabetes was present in 20.6% of 3101 patients with AF and was associated with older age, higher CHA_2_DS_2_-VASc score (4.6 vs. 2.9), more permanent AF (21.5% vs. 16.0%), worse AF-related quality of life, lower use of electrical cardioversion (16.2% vs. 24.6%) and catheter ablation (3.3% vs. 8.6%), and higher 1-year all-cause mortality (11.9% vs. 4.9%) [128]. Risk-factor management should therefore be regarded as an integral component of AF care, although the extent of its disease-modifying effect remains to be established. In ARREST-AF, structured management of weight, blood pressure, glycaemia, lipids, sleep-disordered breathing, and lifestyle factors independently predicted arrhythmia-free survival after ablation (HR 4.8, 95% CI 2.04–11.4), while in REVERSE-AF, ≥10% weight loss was associated with 86% AF freedom and 88% regression from persistent AF to paroxysmal/no AF [129,130]. However, recent randomized evidence in older patients adds necessary nuance: in LOSE-AF, an 8-month low-calorie diet in patients aged 60–85 years with persistent AF achieved greater weight loss than usual care (9.7% vs. 3.1%) without safety concerns, but did not significantly improve AF symptoms, AF burden, cardiac remodelling, or subsequent rhythm-control interventions, suggesting that weight loss should be individualized in older frail-prone patients rather than applied as a uniform therapeutic lever [131]. In evidentiary terms, risk-factor and comorbidity management is guideline endorsed; ARREST-AF and REVERSE-AF provide observational support, whereas LOSE-AF offers randomized evidence that tempers expectations regarding a uniform rhythm benefit from weight loss alone.

Antidiabetic medication selection should account for cardiorenal and rhythm effects. In patients with diabetes and pre-existing AF, SGLT2 inhibitors were associated with lower AF-related healthcare utilization than DPP4 inhibitors (8.7% vs. 10.0%; adjusted HR 0.73, 95% CI 0.55–0.96), and empagliflozin reduced cardiovascular death or HF hospitalization in EMPA-REG OUTCOME participants with baseline AF (HR 0.58, 95% CI 0.36–0.92) [132,133]. Post-ablation data provide additional, although still evolving, evidence for this class: tofogliflozin reduced AF recurrence versus anagliptin in a randomized T2D cohort (24% vs. 47%), and a 2024 meta-analysis of 5623 post-ablation patients found lower AF recurrence with SGLT2 inhibitors (OR 0.45, 95% CI 0.31–0.66) [134,135]. Metformin was associated with greater arrhythmia-free survival after ablation in diabetic patients (55% vs. 40%; adjusted HR 0.66), whereas thiazolidinedione evidence remains mixed: pooled data suggested lower AF risk, but pioglitazone did not reduce recurrence after electrical cardioversion despite lowering inflammatory markers [136,137,138]. GLP-1 receptor agonists may be clinically relevant in obese AF phenotypes; in the LEAF randomized study, adding liraglutide to risk-factor modification before ablation did not significantly enhance early weight or left atrial epicardial adipose tissue reduction, but was associated with higher 1-year freedom from AF/atrial flutter (81% vs. 54%), while a 2026 meta-analysis reported lower post-ablation recurrence with GLP-1 receptor agonist therapy (HR 0.78, 95% CI 0.61–0.99) [139,140,141]. Glycaemic stability may also matter beyond mean HbA1c: among 8790 anticoagulated AF patients, greater HbA1c variability was independently associated with ischemic stroke/systemic embolism and all-cause mortality, including in those with diabetes (HR 1.65 and 1.24, respectively) [142]. Overall, the AF-specific evidence for glucose-lowering therapies is heterogeneous. Data for SGLT2 inhibitors and GLP-1 receptor agonists derive from a mixture of randomized trials, secondary or subgroup analyses, meta-analyses, and observational cohorts, whereas the post-ablation signal for metformin is observational. These agents should therefore be selected primarily according to established glycaemic, cardiovascular, and renal indications; any potential rhythm benefit should be regarded as complementary and, in several clinical settings, hypothesis-generating.

Accordingly, CGM may be used when otherwise indicated for diabetes care, particularly in insulin-treated patients, those with recurrent hypoglycaemia, or patients in whom HbA1c does not adequately reflect daily glucose profiles [76,98]. A higher TIR and lower glycaemic variability are reasonable metabolic objectives, but no AF-specific TIR target or variability threshold has been validated. Selection of glucose-lowering therapy should continue to be based on established glycaemic, weight, cardiovascular, renal, and safety indications; any additional rhythm benefit specifically mediated by reduced glycaemic variability remains unproven.

Stroke prevention remains mandatory in diabetic patients with clinical AF [143,144]. OAC should be guided by thromboembolic and bleeding risk rather than AF pattern, with DOACs preferred over warfarin except in mechanical valves or moderate-to-severe mitral stenosis. In a study-level meta-analysis of phase III AF trials including 18,134 patients with diabetes, DOACs reduced stroke/systemic embolism versus warfarin (RR 0.80, 95% CI 0.68–0.93) and vascular death (RR 0.83, 95% CI 0.72–0.96), without evidence that diabetes attenuated benefit [145]. Bleeding risk still requires active mitigation: among 44,793 rivaroxaban-treated patients with non-valvular AF, major bleeding was higher in those with diabetes than without diabetes (3.68 vs. 2.51 per 100 person-years), although fatal bleeding was rare [146]. For patients unsuitable for long-term OAC, LAAO may be considered selectively. In the NCDR LAAO Registry of 36,681 Watchman recipients, 1-year ischemic stroke, ischemic stroke/systemic embolism, mortality, and major bleeding rates were 1.53%, 2.19%, 8.52%, and 6.93%, respectively; in a national database of 62,220 LAAC procedures, diabetes was not associated with higher overall in-hospital adverse events, although acute kidney injury was more frequent (3.75% vs. 1.96%) [147,148]. PRAGUE-17 supports LAAO as an option in carefully selected high-risk patients, showing noninferiority to DOACs over long-term follow-up and lower nonprocedural clinically relevant bleeding (sHR 0.55) [149]. The anticoagulation recommendations are guideline based and supported by randomized phase III trial evidence. By contrast, the bleeding and national LAAC registry findings are observational, whereas PRAGUE-17 provides randomized comparative evidence for LAAO in carefully selected high-risk patients. Current ESC guidance does not apply sex-specific thresholds for anticoagulation: the 2024 guideline adopts the CHA_2_DS_2_-VA score, which excludes female sex as an independent score component, with OAC considered at a score of 1 and recommended at a score of ≥2 in both women and men [9]. Sex may nevertheless influence the clinical context in which thromboembolic and bleeding risks are expressed, and individualized assessment remains essential.

Rate and rhythm control should be individualized according to symptoms, AF burden, ventricular rate, HF status, left atrial remodelling, frailty, and patient preference Diabetes may complicate rhythm control because it is associated with atrial substrate disease, glycaemic instability, and lower durability of sinus rhythm: in T2D patients undergoing electrical cardioversion, immediate success was lower than in controls (66.6% vs. 84.3%), and maintenance of sinus rhythm at approximately 75 days was also lower (37.2% vs. 61.8%); higher HbA1c, larger left atrial size, lower left ventricular ejection fraction, digoxin use, and T2D predicted failure or relapse [150]. Experimental work also suggests that diabetes may reduce the antiarrhythmic efficacy of sodium-, potassium-, and calcium-channel blockers, emphasizing the need for careful drug selection and monitoring in diabetic electrophysiological substrates [151]. Catheter ablation remains effective but should be embedded within risk-factor control. In a randomized study of 70 patients with T2D and drug-refractory AF, pulmonary vein isolation achieved 80% freedom from AF at 1 year compared with 42.9% with antiarrhythmic drug therapy, with improved quality of life and fewer hospitalizations [152]. A meta-analysis of 15 studies including 1464 diabetic patients reported an overall complication rate of 3.5% and sinus-rhythm maintenance of 66%, but higher age, BMI, and baseline HbA1c predicted recurrence. The markedly higher complication rate reported in the early single-centre study (29.0% vs. 8.2%) should therefore be regarded as a probable outlier, potentially reflecting its limited sample size, earlier procedural era, centre-specific practice, and differences in complication definitions. The contemporary meta-analytic estimate of 3.5% is likely more representative of current practice, consistent with the US Nationwide Inpatient Sample analysis, in which diabetes was associated primarily with longer length of stay rather than an independent excess of procedural complications, whereas obesity more strongly predicted adverse in-hospital outcomes [153,154,155]. Finally, evaluation should move beyond binary recurrence. In DECAAF II, ablation reduced AF burden even among patients classified as having recurrence (65% to 15%), and burden reduction correlated with symptom improvement; however, diabetes and severe atrial fibrosis predicted less improvement [156]. Thus, follow-up in diabetic AF should reassess AF burden, symptoms, OAC appropriateness, renal function, albuminuria, glycaemic variability, HF status, body weight, sleep apnoea, bleeding risk, and rhythm-control candidacy at regular intervals, with treatment escalation driven by evolving substrate and net clinical benefit rather than by rhythm labels alone. Within this hierarchy, individualized rate- and rhythm-control decisions are guideline based; the cardioversion and administrative-database findings are observational, the antiarrhythmic-drug observations are preclinical, and the direct comparison of catheter ablation with drug therapy provides randomized evidence. Sex-specific evidence in diabetes remains sparse. In a national analysis of patients hospitalized with AF and T2D, women were older, had a greater comorbidity burden, and underwent ablation or pacemaker implantation less frequently than men [157]. In the broader CABANA randomized population, ablation reduced recurrent AF in both women and men, although the relative effect was numerically smaller in women (HR 0.64 vs. 0.48; interaction *p* = 0.060), with no significant sex interaction for major clinical outcomes or adverse events [158]. These findings support equitable referral for rhythm-control evaluation while recognizing that women may present later and with a more advanced clinical substrate.

## 6. Evidence Gaps and Future Directions

Important uncertainties remain in the management of AF in patients with diabetes. Although epidemiological and experimental studies consistently implicate atrial fibrosis, autonomic disturbance, lipotoxicity, inflammation, glycaemic variability, and electrical remodelling, the clinical literature is still dominated by observational data. Moreover, the contribution of diabetes itself is often difficult to distinguish from that of obesity, hypertension, CKD, HF, OSA, and ageing. Prospective studies should therefore combine continuous rhythm monitoring with atrial imaging, fibrosis assessment, EAT quantification, metabolomic profiling, circulating biomarkers, and continuous glucose measurements. Such an approach may allow clinically relevant endotypes of diabetic atrial cardiomyopathy to be identified [158]. The prognostic significance of HbA1c variability also warrants further evaluation, particularly in view of recent associations with ischemic stroke/systemic embolism and mortality among anticoagulated patients with AF. Future studies should prespecify sex-stratified analyses and enroll sufficient numbers of women to determine whether diabetic atrial cardiomyopathy phenotypes, screening yield, the benefit-risk balance of anticoagulation, and rhythm-control outcomes differ by sex or menopausal status. Future studies should combine CGM with continuous rhythm monitoring to determine whether changes in TIR or glucose coefficient of variation precede changes in AF burden and whether interventions directed at glycaemic stability improve rhythm outcomes independently of mean HbA1c.

Future studies should also prespecify analyses according to diabetes type, duration, mean glycaemic control, glycaemic variability or hypoglycaemia, and the presence of renal and microvascular complications, rather than combining these biologically distinct exposures under a single diabetes category.

Digital technologies may provide an additional means of refining risk assessment in this population. AI-based models that integrate clinical characteristics with ECG features, atrial imaging, metabolic indices, and circulating biomarkers could improve prediction beyond conventional risk scores. Data obtained from wearable devices may also yield dynamic digital biomarkers of rhythm instability, AF burden, physical activity, and autonomic function [159]. These tools are particularly attractive in high-risk patients with diabetes, in whom intermittent or asymptomatic AF may otherwise remain undetected. Their clinical value, however, will need to be established in prospective studies that assess discrimination, calibration, external validity, and effects on patient outcomes.

The management of subclinical AF in diabetes remains an area of considerable uncertainty. Importantly, device-detected atrial high-rate episodes should not be regarded as equivalent to ECG-confirmed clinical AF, as they may be brief, infrequent, or incompletely adjudicated atrial tachyarrhythmias. In ARTESIA, apixaban reduced stroke or systemic embolism compared with aspirin in patients with device-detected subclinical AF, but this benefit was accompanied by a higher incidence of major bleeding. By contrast, NOAH-AFNET 6 [122] did not demonstrate a favourable net clinical benefit of edoxaban in patients with atrial high-rate episodes. Taken together, these findings do not support routine anticoagulation for all device-detected episodes. Rather, treatment decisions should incorporate episode duration and cumulative AF burden, confirmation of the underlying rhythm, thromboembolic risk, CKD and albuminuria, bleeding susceptibility, frailty, and patient preference. The balance between benefit and harm may be particularly uncertain in patients with a low arrhythmia burden, advanced kidney disease, or a high baseline risk of bleeding.

Dedicated rhythm-outcome trials of cardiometabolic therapies are also required. SGLT2 inhibitors and GLP-1 receptor agonists have been associated with less AF-related healthcare use, lower recurrence after ablation, and more favourable rhythm outcomes, yet the available evidence remains heterogeneous and is frequently derived from observational studies [160,161]. Future rhythm-control trials should also move beyond binary definitions of recurrence. AF burden, symptoms, quality of life, HF events, renal outcomes, and cognitive function may provide a more clinically informative assessment of treatment benefit, either as co-primary or hierarchically ordered endpoints. This approach is supported by the burden analyses from DECAAF II and by secondary findings from EAST-AFNET 4 suggesting that the benefit of early rhythm control is maintained in patients with diabetes.

As this review is a narrative synthesis, several conclusions rely on observational, post hoc, or mechanistic evidence rather than dedicated randomized trials; the principal limitations of the current evidence and corresponding research priorities are summarized in Table 4.

## 7. Conclusions

Diabetes and AF are closely interconnected within a broader cardiometabolic phenotype in which dysglycaemia, obesity, insulin resistance, renal dysfunction, autonomic impairment, inflammation, oxidative stress, fibrosis, lipotoxicity, and electrical remodelling may collectively increase atrial vulnerability. Epidemiological studies consistently associate diabetes with higher incident AF risk and worse downstream outcomes, including HF, CKD progression, thromboembolism, cognitive decline, and mortality. However, the independent contribution of diabetes is difficult to separate from accompanying risk factors such as obesity, hypertension, CKD, HF, OSA, and ageing. Accordingly, diabetic atrial cardiomyopathy should currently be regarded as a descriptive framework for overlapping metabolic, structural, electrical, and autonomic abnormalities, rather than as a validated diagnostic entity.

Management should therefore extend beyond rhythm control alone and follow an integrated, stage-specific AF-CARE strategy. In individuals at increased AF risk, priorities include cardiometabolic risk-factor modification, selective rhythm surveillance, and use of antidiabetic therapies with proven cardiorenal benefit when clinically indicated. In subclinical AF, decisions should integrate AF burden, thromboembolic risk, bleeding risk, renal function, frailty, and patient preference. In clinical AF, stroke prevention, symptom-directed rate or rhythm control, management of obesity, HF, CKD and OSA, and dynamic reassessment of AF burden and treatment benefit remain central. Future studies should incorporate AF burden, substrate characterization, glycaemic variability, cardiorenal outcomes, and patient-centred endpoints to support more precise management of AF across the diabetic disease continuum.

## Figures and Tables

**Table 1:** Key Epidemiological Studies Examining the Relationship Between Diabetes Mellitus and Atrial Fibrillation.

Study	Population	Sample Size	Follow-Up Duration	Main AF-Related Findings
Framingham Heart Study [15]	Community-based adults aged 55–94 years without AF at baseline	4731 (2090 men and 2641 women)	Up to 38 years	Diabetes independently predicted incident AF, with adjusted ORs of 1.4 in men and 1.6 in women. Its population-level contribution was smaller than that of hypertension, HF, or valvular heart disease.
ARIC [16]	Community-based adults without AF at baseline	13,025	Mean 14.5 years	T2D was associated with a 35% higher risk of incident AF (HR 1.35, 95% CI 1.14–1.60). Among participants with diabetes, each 1% increase in HbA1c was associated with higher AF risk (HR 1.13, 95% CI 1.07–1.20).
AMORIS [17]	Swedish adults without baseline CVD who underwent fasting glucose assessment	294,057	Mean 19.1 years	Impaired fasting glucose, undiagnosed diabetes, and diagnosed diabetes were associated with progressively higher AF risks (HRs 1.19, 1.23, and 1.30, respectively). After adjustment for BMI, the association remained significant only for diagnosed diabetes.
FinACAF [21]	Nationwide Finnish cohort of patients with incident AF between 2007 and 2018	229,565	Mean 4.0 years	The prevalence of diabetes among patients with AF increased from 15.5% to 26.3%. Diabetes remained independently associated with ischemic stroke (adjusted IRR 1.22, 95% CI 1.17–1.26) and mortality (adjusted IRR 1.32, 95% CI 1.29–1.34).
Swedish National Diabetes Register T1D study [24]	Individuals with T1D and five age-, sex-, and county-matched population controls per patient	216,238 (36,258 with T1D and 179,980 controls)	Median 9.7 years in T1D and 10.2 years in controls	T1D was associated with higher AF risk in men (HR 1.13) and particularly in women (HR 1.50). Excess risk increased with poorer glycaemic control and renal complications.
ATRIA [27]	Patients with AF and diabetes who were not receiving anticoagulation	2101	Mean 2.5 years	Diabetes duration ≥3 years was associated with increased ischemic stroke risk (HR 1.74, 95% CI 1.10–2.76), whereas HbA1c categories were not independently associated with stroke risk.
ACCORD AF analysis [42]	Adults with T2D randomized to intensive or standard glycaemic control	10,082	Median 4.68 years	Intensive glycaemic control did not reduce incident AF compared with standard treatment. New-onset AF was associated with higher risks of all-cause mortality (HR 2.65), myocardial infarction (HR 2.10), and HF (HR 3.80).
ORBIT-AF [35]	Patients with incident or prevalent AF enrolled in a prospective US outpatient registry	9749; 2874 (29.5%) had diabetes	2 years	Diabetes was associated with poorer AF-related quality of life and higher risks of mortality and hospitalization. It was not independently associated with thromboembolic events, bleeding-related hospitalization, incident HF, or AF progression.
Swiss-AF [36]	Patients with documented AF enrolled in a prospective multicentre Swiss cohort	2411	Cross-sectional baseline analysis	Diabetes was not associated with non-paroxysmal AF but was associated with less frequent symptom perception, poorer quality of life, cognitive impairment, and a greater burden of hypertension, myocardial infarction, HF, and previous stroke.
NOMED-AF [37]	Representative Polish population aged ≥65 years undergoing prolonged wearable ECG monitoring	3014; 881 had diabetes	Cross-sectional assessment; approximately 22 days of ECG monitoring	AF was detected in 25% of participants with diabetes versus 17% without diabetes. Silent AF occurred in 9% versus 7%, and persistent or permanent AF in 12.2% versus 6.9%, respectively.
UK Biobank T2D cohort [39]	Adults with T2D who were free of CVD and CKD at baseline	16,551	Median approximately 11 years	Incident AF was associated with subsequent ASCVD (HR 1.85), HF (HR 4.40), CKD (HR 1.68), all-cause mortality (HR 2.91), and cardiovascular mortality (HR 3.75).

Abbreviations: AF, atrial fibrillation; ARIC, Atherosclerosis Risk in Communities; ASCVD, atherosclerotic cardiovascular disease; BMI, body mass index; CI, confidence interval; CKD, chronic kidney disease; CVD, cardiovascular disease; HbA1c, glycated haemoglobin; HF, heart failure; HR, hazard ratio; IRR, incidence rate ratio; OR, odds ratio; T1D, type 1 diabetes; T2D, type 2 diabetes.

**Table 2:** Proposed mechanisms linking diabetes to atrial fibrillation: evidence base and relevance to human disease.

Mechanistic Domain	Evidence Base	Relevance to Human AF
Fibrotic and structural remodelling	Rodent models and small human atrial-tissue studies support AGE–RAGE, TGF-β/Smad, RAAS, and fibroblast-mediated matrix deposition [41,43,44,45,46,47,48,49].	Fibrosis plausibly promotes conduction heterogeneity, but a diabetes-specific fibrotic phenotype has not been prospectively validated.
Oxidative stress, inflammation, and mitochondrial dysfunction	Experimental studies implicate ROS, NF-κB, IL-1β, oxidized CaMKII, TXNIP, and NLRP3; human tissue shows redox and mitochondrial abnormalities [44,45,48,49,50,51].	Human causality and the rhythm benefit of pathway-specific treatment remain unproven.
Electrical remodelling and calcium handling	Diabetic animal models show connexin loss, conduction slowing, ion-channel changes, RyR2 Ca^2+^ leak, and increased AF inducibility; human studies show P-wave and electromechanical abnormalities [47,50,52,53,54,55,56,57,58,59,60,61].	Clinical findings are consistent with electrical heterogeneity, but direct human ionic evidence is limited.
Autonomic remodelling	Animal studies demonstrate altered sympathetic and parasympathetic responses; clinical evidence includes reduced heart-rate variability, silent AF associations, and cardiovascular autonomic neuropathy [62,63,64,65,66,67,68,69].	Autonomic dysfunction may facilitate AF, although evidence is mainly observational.
Glycaemic variability and hypoglycaemia	Experimental glucose fluctuation increases fibrosis and AF susceptibility; observational studies link HbA1c variability, hypoglycaemia, and perioperative variability with AF [42,70,71,72,73,74,75].	No AF-specific CGM target or proof that reducing variability improves rhythm outcomes exists.
Obesity, insulin resistance, and EAT	Human EAT transcriptomic, secretome, extracellular-vesicle, and microRNA studies are supported by obesity models showing inflammatory, fibrotic, and electrical remodelling [51,76,77,78,79,80,81,82,83,84,85].	EAT is clinically relevant but difficult to separate from generalized obesity and comorbidity burden.
Lipotoxicity and impaired substrate utilisation	Human imaging and atrial-tissue studies show steatosis and impaired oxidation; experimental restoration of fatty-acid oxidation or AMPK signalling reduces AF susceptibility [45,60,86,87,88,89,90,91,92].	Independent prognostic and therapeutic relevance in human AF remains uncertain.
Obstructive sleep apnoea	Experimental studies implicate intermittent hypoxaemia, pressure swings, autonomic instability, oxidative stress, Ca^2+^ leak, and connexin loss; human T2D cohorts show higher AF risk [82,93,94].	OSA may amplify the diabetic atrial substrate, but diabetes-specific treatment trials are limited.

Abbreviations: AF, atrial fibrillation; AGE, advanced glycation end product; AMPK, AMP-activated protein kinase; CGM, continuous glucose monitoring; EAT, epicardial adipose tissue; IL-1β, interleukin-1 beta; NLRP3, NOD-like receptor family pyrin domain-containing 3; OSA, obstructive sleep apnoea; RAAS, renin–angiotensin–aldosterone system; ROS, reactive oxygen species; T2D, type 2 diabetes; TGF-β, transforming growth factor beta.

**Table 3:** Summary of therapeutic strategies for AF in diabetes.

Therapeutic Domain	Practical Strategy	Main Clinical Role	Key Limitations
Cardiometabolic risk-factor control	Optimize weight, BP, glycaemia, CKD, HF, OSA, exercise, alcohol intake, and lifestyle.	Foundational therapy across all AF stages.	Broad clinical benefit; AF-specific effect varies by intervention.
Glucose-lowering therapy	Use metformin, SGLT2 inhibitors, GLP-1 receptor agonists, or related agents according to glycaemic, weight, cardiorenal, and safety indications.	May improve cardiometabolic substrate and possibly reduce AF susceptibility or recurrence.	Rhythm benefit is complementary and not the primary indication.
Rhythm surveillance	Opportunistic ECG; selective Holter, patch, or wearable monitoring in older, symptomatic, or high-risk patients.	Detects silent or early AF in enriched-risk diabetes phenotypes.	Universal diabetes-specific AF screening is not established.
Stroke prevention in clinical AF	Use OAC according to thromboembolic risk; prefer DOACs unless contraindicated.	Reduces stroke/systemic embolism in eligible patients with AF.	Modify bleeding risk rather than using it alone to deny OAC.
Subclinical AF/AHRE	Confirm rhythm, quantify AF burden, and individualize OAC decisions.	Guides management of device- or wearable-detected AF.	AF-burden threshold for OAC remains uncertain.
Rate control	Use symptom-guided ventricular rate control when rhythm control is not required or not feasible.	Improves symptoms and prevents tachycardia-related deterioration.	Does not directly modify AF substrate or progression.
Rhythm control	Consider cardioversion, antiarrhythmic drugs, or catheter ablation according to symptoms, AF burden, HF, substrate, and preference.	Reduces symptoms and AF burden; may improve outcomes in selected patients.	Diabetes, obesity, CKD, OSA, and atrial substrate may affect success.
LAAO	Consider in selected high-risk patients unsuitable for long-term OAC.	Alternative stroke-prevention strategy when OAC is problematic.	Requires careful procedural and long-term risk assessment.
Longitudinal reassessment	Reassess AF burden, symptoms, OAC indication, renal function, bleeding risk, HF, weight, OSA, glycaemia, adherence, and rhythm-control candidacy.	Keeps treatment aligned with changing risk and disease course.	Management should remain dynamic rather than one-time.

Abbreviations: AF, atrial fibrillation; AHRE, atrial high-rate episode; BP, blood pressure; CKD, chronic kidney disease; DOAC, direct oral anticoagulant; ECG, electrocardiogram; GLP-1, glucagon-like peptide-1; HF, heart failure; LAAO, left atrial appendage occlusion; OAC, oral anticoagulation; OSA, obstructive sleep apnea; SGLT2, sodium-glucose cotransporter-2.

**Table 4:** Principal Evidence Gaps and Priorities for Future Research in Atrial Fibrillation and Diabetes.

Research Domain	Key Unresolved Question	Current Evidence Limitations	Suggested Future Study Design
AF screening in diabetes	Which diabetic populations derive clinically meaningful benefit from systematic or prolonged AF screening, and what monitoring duration and modality are optimal?	Available studies are heterogeneous in age, baseline risk, monitoring technology, and AF definition. Increased AF detection has not consistently translated into reductions in stroke, HF, or mortality, and diabetes-specific randomized evidence is limited.	Pragmatic randomized trials comparing usual care with risk-enriched screening strategies using intermittent ECG, patch monitors, or wearables; outcomes should include AF detection, anticoagulation uptake, stroke, bleeding, HF events, quality of life, and cost-effectiveness.
Glycaemic variability	Does short- or long-term glycaemic variability independently contribute to incident AF, AF burden, or adverse outcomes beyond mean HbA1c?	Most evidence is observational and relies on heterogeneous indices of variability. Residual confounding by disease severity, treatment intensity, hypoglycaemia, CKD, and comorbidity is substantial, while continuous glucose monitoring data remain sparse.	Prospective cohorts with simultaneous continuous glucose and rhythm monitoring, followed by randomized intervention studies targeting glycaemic stability rather than HbA1c alone.
Antidiabetic therapies and AF outcomes	Do SGLT2 inhibitors, GLP-1 receptor agonists, metformin, or newer incretin-based therapies directly reduce AF incidence, burden, or progression?	AF is rarely a prespecified primary endpoint in cardiometabolic trials. Existing estimates are often derived from post hoc analyses, adverse-event reporting, observational comparisons, or heterogeneous meta-analyses, limiting causal inference.	Adequately powered randomized trials with prespecified rhythm endpoints, standardized AF ascertainment, continuous or repeated monitoring, and stratification by obesity, HF, CKD, and baseline AF status.
Post-ablation recurrence	Can cardiometabolic treatment improve rhythm outcomes after AF ablation in patients with diabetes, and which phenotypes benefit most?	Most studies are retrospective or non-randomized, use inconsistent recurrence definitions and monitoring intensity, and inadequately account for weight change, glycaemic control, atrial fibrosis, AF burden, and concomitant risk-factor modification.	Multicentre randomized trials of structured cardiometabolic interventions initiated before and continued after ablation, with continuous rhythm monitoring and endpoints including AF burden, repeat ablation, symptoms, HF events, and quality of life.
Anticoagulation for subclinical AF	Which patients with diabetes and device-detected atrial high-rate episodes or subclinical AF achieve a favourable net clinical benefit from anticoagulation?	ARTESIA and NOAH-AFNET 6 produced differing estimates of benefit and harm. Uncertainty is greatest in patients with low AF burden, advanced CKD, frailty, or high bleeding risk, and device-detected episodes are not equivalent to ECG-confirmed clinical AF.	Individual-participant-data analyses and dedicated randomized trials stratified by episode duration, cumulative AF burden, ECG confirmation, renal function, albuminuria, frailty, bleeding risk, and prior stroke.
Cognitive outcomes	Does AF prevention, earlier detection, or rhythm control reduce cognitive decline and dementia in patients with diabetes?	Evidence is predominantly observational, cognitive assessment is inconsistent, and the relative contributions of silent cerebral infarction, hypoperfusion, vascular disease, anticoagulation, and glycaemic injury remain uncertain.	Long-term prospective studies and randomized trials incorporating standardized cognitive testing, brain imaging, AF burden, anticoagulation exposure, and adjudicated dementia outcomes.
Diabetic atrial cardiomyopathy phenotyping	Can reproducible biological and imaging endotypes identify patients at risk of AF onset, progression, or treatment failure?	No validated clinical definition or diagnostic standard exists. Current studies are small and use heterogeneous combinations of atrial imaging, fibrosis markers, EAT measures, ECG indices, metabolomics, and circulating biomarkers.	Large, deeply phenotyped longitudinal cohorts integrating ECG, continuous rhythm data, echocardiography, CT or CMR, EAT and fibrosis assessment, metabolomics, proteomics, inflammatory biomarkers, and continuous glucose metrics, followed by external validation.

Abbreviations: AF, atrial fibrillation; CKD, chronic kidney disease; CMR, cardiovascular magnetic resonance; CT, computed tomography; EAT, epicardial adipose tissue; ECG, electrocardiography; HF, heart failure.

## Data Availability

No new data were created or analyzed in this study.

## Abbreviations

AF, atrial fibrillation; BP, blood pressure; CHA_2_DS_2_-VA, congestive heart failure, hypertension, age ≥ 75 years, diabetes, prior stroke/transient ischemic attack/systemic embolism, vascular disease, and age 65–74 years; CHA_2_DS_2_-VASc, congestive heart failure, hypertension, age ≥ 75 years, diabetes, prior stroke/transient ischemic attack/systemic embolism, vascular disease, age 65–74 years, and sex category; CKD, chronic kidney disease; DOAC, direct oral anticoagulant; ECG, electrocardiogram; HF, heart failure; LA, left atrial; OAC, oral anticoagulation; OSA, obstructive sleep apnea; TIA, transient ischemic attack.
